# The NARCOguide index – a novel parameter for monitoring depth of hypnosis during anaesthesia/sedation with propofol: A comparison study with the Narcotrend index

**DOI:** 10.1097/EA9.0000000000000057

**Published:** 2024-07-18

**Authors:** Florian Hetzer, Stefan Horack, Gert Küchler, Jens Broscheit

**Affiliations:** From the SOMNOmedics AG, Am Sonnenstuhl 63, 97236 Randersacker, Germany (FH, GK), the Kompetenzzentrum Anästhesiologie, Untere Marktstr. 56, 96515 Sonneberg, Germany (SH), the Universitätsklinik für Neurologie, Inselspital, Freiburgstrasse (Rosenbühlgasse 25), 3010 Bern, Switzerland

## Abstract

**BACKGROUND:**

The NARCOguide algorithm calculates an EEG-derived index to monitor the hypnotic component of anaesthesia.

**OBJECTIVE:**

This study evaluates the accuracy of the index calculated by NARCOguide against the Narcotrend index as a reference. Secondly, the automatic detection of burst-suppression patterns as represented by the burst suppression ratio was compared.

**DESIGN:**

Comparative study to assess the agreement between two medical devices.

**SETTING:**

At two study centres, patient data were collected from a total of 40 adults receiving general anaesthesia or sedation with propofol.

**INTERVENTIONS:**

Patients underwent either general anaesthesia for oral surgery with propofol/remifentanil/rocuronium (study centre 1) or light general anaesthesia/deep sedation with propofol alone for laryngoscopic upper airway exploration (study centre 2).

**MAIN OUTCOME MEASURES:**

In a posthoc analysis, the NARCOguide index was compared with the Narcotrend index. Comparison was made after averaging over 1 min at defined clinical markers using classic linear least squares regression and Bland–Altman plots. Precision and recall for the detection of burst suppression were determined using human scoring as a reference.

**RESULTS:**

Data analysis showed good agreement [Bland–Altman mean difference (MD) = −2.3; limits of agreement = −27.1, to +22.4; *n* = 1209] and high correlation (*r*^2^ = 0.76) between the depth of anaesthesia index calculated by NARCOguide and Narcotrend. The precision and recall of NARCOguide and Narcotrend for the detection of burst suppression were in a similar range. Over the entire dataset, the NARCOguide algorithm showed higher precision and recall than the Narcotrend algorithm (56% vs. 36% and 68% vs. 58%, respectively).

**CONCLUSION:**

The NARCOguide index can be used to monitor the hypnotic component of anaesthesia in patients undergoing general anaesthesia or sedation with propofol, with a performance similar to that of the Narcotrend index.

**TRIAL REGISTRATION:**

Trial registration number: 18020, regulatory authority: Ethikkommission der bayerischen Landesärztekammer, chairman: Dr med. Gerald Quitterer, applicant: Dr Gert Küchler, date of approval: 12. Jun 2018, completion of data collection: 12 December 2018, study completion: 31 March 2022.

## Graphical abstract

**Figure:**
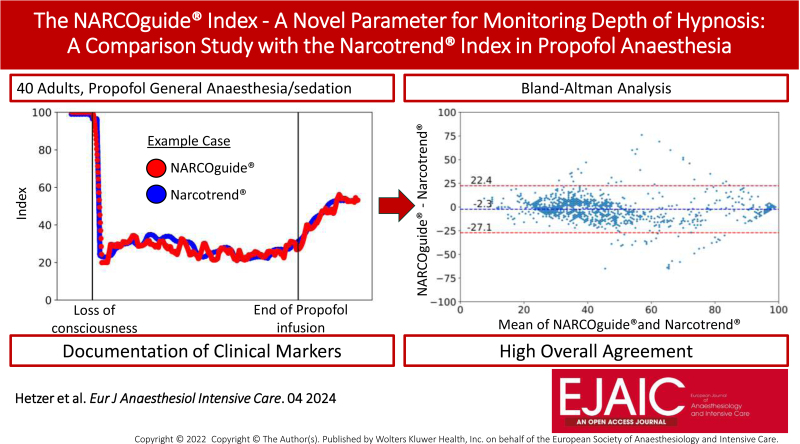

**KEY POINTS**

- The NARCOguide algorithm calculates an EEG-derived index to monitor the hypnotic component of anaesthesia.
- This study compares the accuracy of the index calculated by NARCOguide with that of the Narcotrend.
- The “*depth of anaesthesia*” index calculated by NARCOguide and Narcotrend showed good agreement (Bland–Altman mean difference = −2.3; limits of agreement = −27.1 to +22.4; *n* =1209) and high correlation (*r*^2^ = 0.76).
- The precision and recall of NARCOguide and Narcotrend for the detection of burst suppression were in a similar range.
- The NARCOguide index can be used to monitor the hypnotic component of general anaesthesia or sedation with propofol with similar performance compared to the Narcotrend index.

## Introduction

For decades, the adequacy of general anaesthesia has been assessed on the basis of anaesthetic concentration and nonspecific clinical parameters such as blood pressure, heart rate, patient responses, sweating, lacrimation or pupil reactivity. Although these parameters are affected by anaesthesia, they do not reflect the effects of anaesthesia on the main target organ, the brain. Today, the majority of anaesthetic depth monitoring devices are based on parameters derived from the electroencephalogram (EEG), which provides insight into brain activity. Anaesthetics cause characteristic, dose-dependent, and reproducible changes in the EEG.^1^

By analysing the EEG, it is possible to determine the hypnotic component of anaesthesia. In this manuscript, the term “*depth of anaesthesia*” (DoA) will be used to refer to the hypnotic component, excluding antinociception and neuromuscular blockade, the other modalities which together form the triad of general anaesthesia.

The NARCOguide algorithm is implemented in the NARCOguide DoA monitor (NEUROdiagnostics, a division of SOMNOmedics AG, Germany, http://www.narcoguide.de/). The NARCOguide monitor records the raw frontal EEG and electrooculogram (EOG) signals from both eyes. These raw signals are filtered and cleaned from nonphysiological artefacts. Artefact detection is based on EEG amplitude to exclude offsets (e.g. mechanical influence), EEG increment to exclude steep ascents (e.g. electromagnetic artefacts from electrocautery devices) and EEG impedance to exclude background noise (e.g. mains frequency 50/60 Hz). The NARCOguide algorithm analyses the EEG and EOG signals in the time and frequency domain (Figure S1, Supplemental Digital Content). The time domain features are burst suppression (BS) patterns and eye movements. Patterns of BS are described quantitatively by the burst suppression ratio (BSR) – a dimensionless index ranging from 0 to 100, corresponding to the ratio of suppression lines within the last 60 s of the EEG signal.

The detection of BS patterns is based on the detection of suppression lines. Burst detection is used as a secondary parameter to distinguish between bursts and suppressions. Bursts and suppressions are defined by high or low root mean square (RMS) of the EEG signal and high or low RMS of the first derivative of the EEG signal, respectively. The absence of bursts is not a limitation to the detection of suppression, as in the case of very long suppression lines (>60 s). An isoelectric EEG is defined as a signal with an RMS of less than 3 μV. In the presence of bursts, suppression detection is less dependent on absolute EEG amplitude, which varies between individuals (e.g. different age groups).

Eye movements detected in the EOG signals contribute to the detection of wakefulness. The frequency domain features are derived from the Fast Fourier Transform (FFT) of the EEG signal in the frequency range of 0.5 to 47 Hz. The FFT is divided into the δ-, θ-, α-, β- and γ spectral bands and signal smoothing is performed. The algorithm includes spectral band correlation functions that are used as input to an empirical rule-based model to determine the NARCOguide index. This index describes the hypnotic component of anaesthesia and ranges from 100 (awake) to 0 (isoelectric EEG). A description of the NARCOguide index scale can be found in the Supplementary Appendix (Figure S2).

In contrast to established *depth of anaesthesia* monitors, the major advantage of the NARCOguide device is that both the haemodynamic (standard monitoring) and the central effects (EEG monitoring) of anaesthetics can be recorded and shown on the screen of one monitoring device. The NARCOguide records the vital parameters SpO_2_, photoplethysmogram (PPG), electrocardiogram (ECG) and heart rate. Pulse transit time (PTT) can be determined from the ECG and PPG. After entering the patient's height and performing a one-point calibration against a cuff-based blood pressure measurement, the noninvasive systolic and diastolic blood pressures can be calculated from the PTT.^2^ These blood pressure values are noninvasive, continuous (beat by beat) and nonreactive (measurement is performed without the audible and mechanical inflation of a cuff). The continuous blood pressure trend calculated by the NARCOguide device can be useful to measure the immediate effect of propofol/remifentanil on blood pressure noninvasively. Blood pressure is a standard parameter used to control anaesthesia.^3^

The aim of the present study was to evaluate the accuracy of the EEG-based DoA index calculated by NARCOguide compared with the Narcotrend index in propofol-based anaesthesia. Secondly, the automatic detection of BS patterns as represented by the BSR was compared

## Methods

After approval of the study by the ethics committee (Trial registration number: 18020, regulatory authority: Ethikkommission der bayerischen Landesärztekammer, chairman: Dr med. Gerald Quitterer, applicant: Dr Gert Küchler, date of approval: 12 June 2018), patients aged ≥18 years who were scheduled to receive propofol anaesthesia or sedation at the study centres were included after written informed consent was obtained. Exclusion criteria were the presence of major neurological or cerebrovascular disease, known allergy to propofol, use of psychotropic drugs, opioids, opiate/drug abuse affecting the EEG, and active implantable stimulators (e.g. pacemakers).

The EEG/EOG were recorded using the SOMNO HD system (SOMNOmedics AG, Germany). The haemodynamic parameters SpO_2_, blood pressure and ECG were also recorded. Postoperatively, using the NARCOguide algorithm, the NARCOguide index and BSR were calculated from the EEG/EOG on a computer.

In preparation for surgery, the electrodes and sensors for the Narcotrend and SOMNO HD devices were applied to the patient according to the application plan (see Figures S3 and S4). The impedance values of both devices were checked and documented. For standard intraoperative monitoring, ECG electrodes, SpO_2_ finger clip and blood pressure cuff were applied.

Data recording on both devices was started at least 1 min before induction of anaesthesia, if allowed by the surgical procedure. Anaesthesia was controlled by the anaesthesiologist based on an assessment of the patient's overall clinical condition, taking into account the display of the Narcotrend monitor.

After administration of midazolam (2.5 mg) and atropine (0.25 mg), propofol was administered in an identical manner in both participating study centres.

In study centre 1, remifentanil, rocuronium and propofol were administered intravenously for oral surgery (OS group). After premedication, an additional intravenous dose of dexamethasone 4 mg was administered. Propofol and remifentanil were administered via a target-controlled infusion (TCI) syringe pump (Schnider model, B. Braun Perfusor Space). After loss of consciousness, rocuronium was given as a single intravenous dose, and 30 to 60 s later nasotracheal intubation was performed to enable mechanical ventilation of the patients’ lungs.

In study centre 2, propofol was administered for light general anaesthesia/procedural sedation (PS group) for laryngoscopic upper airway exploration. In contrast to centre 1, these patients did not experience any painful surgical manipulation during PS. Neither intravenous analgesics nor muscle relaxants were used during the procedures. The endoscope was inserted after topical analgesia (nasal lidocaine spray 10 mg).

In both centres, the anaesthetist documented the time points of the following clinical markers during surgery: bolus of propofol (BoP), loss of consciousness (LoC), end of propofol infusion (EoP), onset of spontaneous respiration after ventilation (BoR), and patient awakening (WoP). No ventilation was performed at study centre 2, therefore BoR was not applicable. BoP was defined as the start of propofol infusion via the TCI pump. LoC was defined by absence of response to verbal request. BoR was defined by the presence of thoracic movements, capnography and flow diagram at study centre 1, and by thoracic movements only in study centre 2.

Measurement was stopped when the patient woke up. Data were exported from both devices to a computer. Later, the SOMNO HD raw EEG/EOG signals were used as inputs for the NARCOguide monitor and its algorithm, which includes filtering, artefact detection and impedance checks (Figure S1, Supplemental Digital Content). Since this was undertaken on a computer after the procedure, the index produced had to be aligned to the time base of the original raw EEG/EOG.

After running the NARCOguide algorithm to create the index values, these values were pooled over 1 min. Pooling involved taking the arithmetic mean of the values 30 s on each side of the relevant time points. Missing data points were treated as gaps. In the absence of any valid value within this interval, the comparison was not possible. For a more focused analysis based on clinical markers, the following clinically relevant time points were used: 1 min before LoC, 1 min after LoC, time of EoP, 1 min before WoP and 1 min after WoP. The index scales of the Narcotrend and NARCOguide devices both range from 0 to 100. The definition of anaesthesia stages of the two indexes/manufacturers differs in number of stages, ranges, and annotation (NARCOguide scale, Figure S2 Narcotrend scale.^4^)

The agreement between NARCOguide and Narcotrend DoA index was assessed using Bland-Altman analysis.^5^ The limits of agreement are defined as the mean difference ± 1.96 standard deviations. The analysed data do not contain replicates nor repeated measurements, all available data pairs were included in the analysis. The correlation between NARCOguide and Narcotrend DoA index was determined by a classical least squares linear regression analysis (LSR). The observed *r*^2^ values were considered statistically significant at *P* ≤ 0.05.

The relative δ (0.5–4 Hz) and α (8–12 Hz) EEG band power was calculated after FFT transformation as the respective band power divided by the total power. Due to the experimental setup of the study, the FFT calculation was performed on the raw EEG signal. This includes artefacts in the raw EEG signal and assumes that the NARCOguide and Narcotrend algorithms analysed identical raw EEG signals. The complete dataset was analysed at two clinically relevant time points: 1 min after loss of consciousness (LoC + 1 min), and during emergence (1 min after end of propofol infusion (EoP + 1 min)) from general anaesthesia. A two-sided *t*-test was performed to compare the average relative δ- and α EEG band powers at the two time points.

The accuracy of the NARCOguide and Narcotrend algorithms in detecting BS was compared with the anaesthesiologist's clinical assessment of the raw EEG signal and between the two algorithms. For this purpose, the raw EEG signal was examined for BS patterns by the anaesthesiologist. The raw EEG signal was divided into 20 s segments for analysis. A total of 99 EEG segments containing BS patterns were identified and used for BSR analysis. Detection of BS was defined as a true positive if a segment contained at least one BS pattern as judged by the anaesthetist and the algorithm simultaneously determined a BSR value >0.

Precision and recall were calculated.^6^ Precision represents the ratio of BS patterns correctly detected by the algorithm [true positives (TP)] to the total of BS patterns detected by the algorithm [TP and false positives (FP)]. Recall represents the ratio of BS patterns correctly detected by the algorithm (TP) to the total of BS patterns scored by the anaesthesiologist [TP and false negatives (FN)]. The *F*1-score was calculated using the formula (2 × precision × recall)/(precision + recall), the accuracy was calculated as (TP + TN)/(TP + FN + TN + FP) and the specificity was calculated as TN/(TN + FP).

## Results

A total of 48 patients were initially enrolled of which 8 were excluded from analysis. This was due to poor EEG signal quality in either the Narcotrend, the SOMNO HD or both devices. Low EEG signal quality made it impossible to calculate the Narcotrend or NARCOguide index and therefore to compare the indices within the study. Thus 40 patients (13 female, 27 male), aged between 18 and 67 years (mean age 42.3 ± 15.0 years) were included in the analysis. Following evaluation of the overall study population, datasets from patients undergoing either procedural sedation (PS) or oral surgery (OS) were analysed separately. The characteristics of the study population, the PS group and the OS group are described in detail in the Supplementary Appendix (Tables S1–S3).

**Fig. 1:**
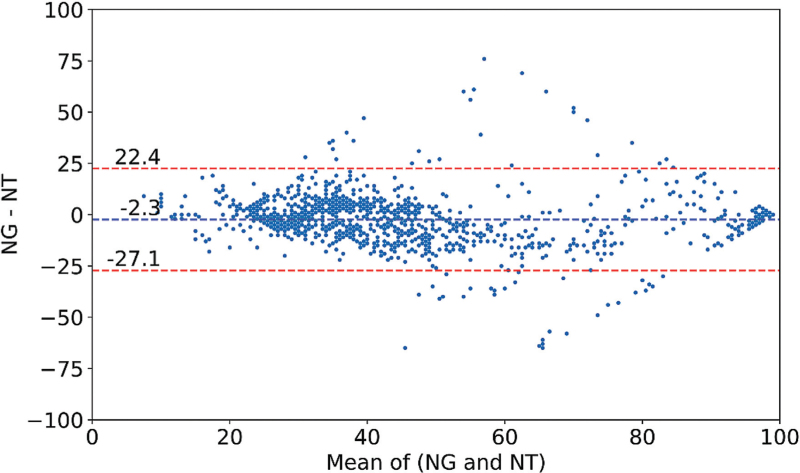
Bland–Altman plot for the NARCOguide and Narcotrend index values over the entire dataset. Individual values averaged over 1 min intervals (*n* = 1209).

**Fig. 2:**
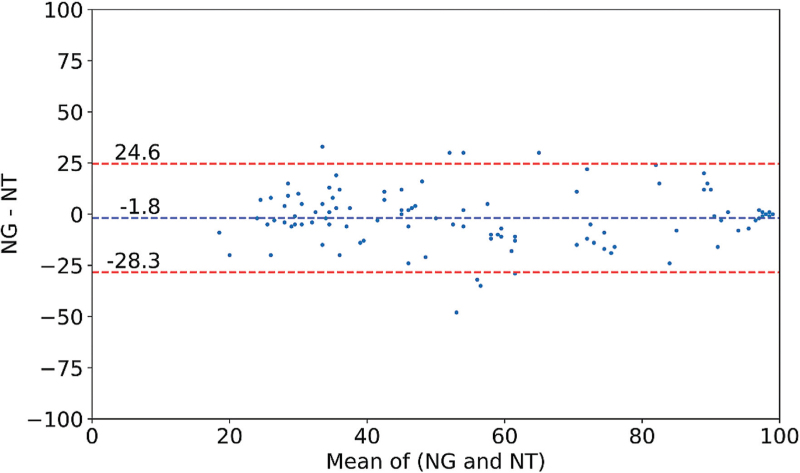
Bland–Altman plot for the NARCOguide and Narcotrend index values based on clinical markers documented by the anesthesiologist over the entire dataset. Individual values averaged over 1 min intervals (*n* = 111).

The results of the Bland–Altman analysis and the LSR are summarised in Table 1. The mean difference across the total dataset was −2.3 with limits of agreement of −27.1 to +22.4 (*n* = 1209) (Fig. 1). The LSR analysis showed a high correlation (*r*^2^ = 0.76). A more focused analysis based on the clinical markers documented by the anaesthetist showed a mean difference of −1.8 with limits of agreement of −28.3 to +24.6 (*n* = 111) (Fig. 2). The LSR analysis showed a high correlation (*r*^2^ = 0.76). The mean difference ranged from −5.9 to +1.3, with the narrowest limits of agreement observed before LoC (LoC –1 min: −18.9 to +17.6). The worst agreement was found after LoC (LoC + 1 min: −34.3 to +32.8).

**Table 1:** Results of Bland–Altman analysis and LSR to assess agreement and correlation between the Narcotrend (NT) index and the NARCOguide (NG) index

Dataset	MD NG–NT	MD -1.96 SD	MD +1.96 SD	*r*^2^ (LSR)	*P*	*N*
Total	−2.3	−27.1	22.4	0.76	≤0.05	1209
Clinical Markers	−1.8	−28.3	24.6	0.76	≤0.05	111
LoC – 1 min	−0.6	−18.9	17.6	0.44	≤0.05	24
LoC + 1 min	−0.7	−34.3	32.8	0.48	≤0.05	27
EoP	+1.3	−23.4	26.2	0.55	≤0.05	34
WoP – 1 min	−4.1	−27.9	19.7	0.56	≤0.05	15
WoP + 1 min	−5.9	−33.8	22.0	0.55	≤0.05	11
OS	−1.8	−23.4	19.6	0.72	≤0.05	730
Clinical Markers	−0.6	−28.3	27.0	0.74	≤0.05	46
LoC – 1 min	+1.2	−23.0	25.6	0.48	≤0.05	11
LoC + 1 min	−2.8	−41.0	35.4	0.52	≤0.05	11
EoP	+3.2	−17.0	23.4	0.72	≤0.05	15
WoP – 1 min	+6.5	−14.9	27.9	0.79	≤0.05	6
WoP + 1 min	−1.6	−26.1	22.7	0.64	ns	3
PS	−2.9	−31.9	26.0	0.72	≤0.05	479
Clinical Markers	−2.7	−28.2	22.7	0.77	≤0.05	65
LoC – 1 min	−2.2	−11.9	7.5	0.07	ns	13
LoC + 1 min	0.6	−28.9	30.3	0.44	≤0.05	16
EoP	0.0	−27.6	27.5	0.37	≤0.05	19
WoP – 1 min	−11.2	−23.7	1.3	0.90	≤0.05	9
WoP + 1 min	−7.5	−36.0	21.0	0.53	≤0.05	8

Results are reported for the total study population (*n* = 40), patients undergoing oral surgery (OS, *n* = 17) and patients undergoing procedural sedation (PS, *n* = 23). For each group, analysis was performed over the entire duration of surgery and on the basis of clinically relevant time points defined by the anaesthesiologist (clinical markers). Clinical markers were defined as 1 min before loss of consciousness (LoC – 1 min); 1 min after LoC (LoC + 1 min); at the end of propofol infusion (EoP); 1 min before waking up of patient (WoP) (WoP – 1 min); and 1 min after WoP (WoP + 1 min). See methods section for a detailed description of data acquisition and analysis. NG, NARCOguide; NT, Narcotrend; MD, mean difference; SD, standard deviation; LSR, linear least squares regression; LoC, loss of consciousness; EoP, end of propofol infusion; WoP, waking up of patient; OS, oral surgery; PS, procedural sedation, significance level *P ≤* 0.05; ns, not significant (*P* > 0.05).

The blue dotted line represents the mean difference between the NARCOguide index and the Narcotrend index, while the red dotted lines represent the limits of agreement (the mean of the difference ±1.96 standard deviations).

The blue dotted line represents the mean difference between the NARCOguide index and the Narcotrend index, while the red dotted lines represent the limits of agreement (the mean of the difference ± 1.96 standard deviations).

For the OS group, the mean difference was −1.8 with limits of agreement of −23.4 to +19.6 (*n* = 730). The LSR analysis showed a high correlation (*r*^2^ = 0.71). A focused analysis based on the clinical markers documented by the anaesthetist showed a mean difference of −0.6 with limits of agreement of −28.3 to +27.0 (*n* = 46). The LSR analysis showed a high correlation (*r*^2^ = 0.74). For the PS group, the mean difference was −2.9 with limits of agreement of −31.9 to +26.0 (*n* = 730). The LSR analysis showed a high correlation (*r*^2^ = 0.72). A focused analysis based on the clinical markers documented by the anaesthetist showed a mean difference of −2.7 with limits of agreement of −28.2 to +22.7 (*n* = 46). Bland–Altman plots for the OS and PS groups are depicted in the Supplementary Appendix (Figures S5–S8).

Overall, the agreement in the OS group is comparable with the PS group (MD = −1.8 vs. −2.8, with limits of agreement of −23.4 to 19.6 vs. −31.9 to 26.0, respectively). The largest discrepancy was observed before WoP in the PS group (WoP – 1 min: MD = −11.2), indicating a tendency of the NARCOguide index to underestimate compared with the Narcotrend.

An example measurement including the NARCOguide and Narcotrend index as well as the relative δ (0.5–4 Hz) and α (8–12 Hz) spectral EEG band power is shown in the Supplementary Appendix (Figure S9). A comparison of the NARCOguide index, Narcotrend index, relative δ-band power and relative α-band power between induction and emergence from anaesthesia is shown in Figure S10. The analysis includes the entire dataset and showed a significant difference between δ and α band power (significance level *P* ≤ 0.05), while no significant difference was found for the NARCOguide and Narcotrend index.

The results of the precision and recall analysis for BS detection are summarised in Table 2. Over the entire dataset, the precision and recall of NARCOguide and Narcotrend BS detection were in a similar range, with the NARCOguide algorithm demonstrating higher precision and recall than the Narcotrend algorithm (56% vs. 36% and 68% vs. 58%, respectively). In the OS group, the same trend was observed (60% vs. 50% and 75% vs. 67%), whereas in the PS group, the Narcotrend algorithm performed better than the NARCOguide algorithm (23% vs. 33% and 21% vs. 40%). The *F*1-score, which takes into account precision and recall values, also reflects the trends described above. The accuracy and specificity values calculated for both devices were above 97% for the entire dataset, above 95% in the OS group and above 98% in the PS group.

**Table 2:** Agreement between the NARCOguide and Narcotrend algorithms for burst suppression (BS) detection compared with the clinical assessment of the raw EEG signal (scored per 20 s EEG segment)

Dataset	Total		OS		PS	
Algorithm	NG	NT	NG	NT	NG	NT
Precision (%)	56	42	60	43	23	33
Recall (%)	68	58	75	62	21	40
*F*1 score (%)	61	49	67	51	22	36
Accuracy (%)	97.9	97.1	97.3	95.9	98.7	98.8
Specificity (%)	98.7	98.0	98.1	97.1	99.4	99.3
*n* (scored BS)	99	99	85	86	14	15
*n* (total)	3975	4693	2322	2494	1653	1757

BS, burst suppression; *n*, number of 20 s EEG-sections; NG, NARCOguide; NT, Narcotrend; OS, oral surgery; PS, procedural sedation.

## Discussion

In this study, after artefact removal, 1209 data pairs were obtained from 40 adults undergoing OS or PS and these were used to compare the relative performance of the NARCOguide index with the Narcotrend index. Overall, the correlation between the NARCOguide index and the Narcotrend index was high (*r*^2^ = 0.76). The mean difference (−2.3) was small with limits of agreement of −27.1 to +22.4. Similar results were found in a study comparing the Bispectral Index (BIS) with State Entropy^7^ and in a study comparing the BIS with the Cerebral State Index.^8^ In the patient groups undergoing OS or PS, the NARCOguide algorithm performed similarly, suggesting broad applicability with little influence from the type of surgery.

Analysis of the dataset was restricted to specific clinically relevant phases during anaesthesia (clinical markers) to further elucidate relative performance (Table 1 and Figure 2). For this purpose, Bland-Altman analysis is a more appropriate method than LSR. Briefly illustrated, both indices can correctly identify a particular patient state by showing values with a narrow distribution (e.g., 95–100 for the awake state). As the distribution of values narrows, small differences are overly penalised by LSR analysis, resulting in a low coefficient of determination (*r*^2^), whereas Bland-Altman analysis indicates high agreement. Therefore, coefficients of determination (*r*^2^) must be considered carefully in this comparison.

As a secondary objective, this study evaluated the relative performance of the NARCOguide algorithm vs. the Narcotrend algorithm for the detection of burst suppression (BS). Over the entire dataset, precision and recall of the NARCOguide algorithm were higher than for the Narcotrend algorithm. In the group of patients undergoing PS, NARCOguide showed the same precision as Narcotrend (23%), whereas the recall was lower (21% vs. 38%). The low performance in this patient group may be partly due to the limited number of scored BS segments (*n* = 14 over two individual measurements) leading to a higher contribution of detection time delay, which is a limitation of this study. According to the Narcotrend scale, burst suppression patterns are associated with index values of 0 to 12 (stage F).^9^ However, we observed that Narcotrend BS detection does not necessarily correspond to the Narcotrend index, as in some measurements we observed a BSR markedly above 0, while the patient was awake (defined by a Narcotrend index of 95–100, stage A). The Narcotrend algorithm calculates the BSR based on the detection of suppression lines associated with low EEG integral (aEEG).^9,10^ EEG amplitudes are similarly low in both the awake and BS states. The absence of typical awake-associated artefacts may explain the moderate performance of Narcotrend.^10^

At this point, the limitations of this study should be addressed. Based on the experimental design of this study, it is not possible to ensure that both monitors analyse identical EEG signals, as the EEG signals are derived from different leads and electrodes positioned at different places/points on the patient's forehead. Secondly, the index values calculated by the two algorithms inherit a certain time delay between the EEG signal and the index. The algorithms have different internal sampling, processing, and smoothing methods. To mitigate this effect, we pooled and extracted data from 1 min intervals. In another respect, the relationship between the actual depth of the hypnotic component of anaesthesia as a function of anaesthetic dose and the calculated index as a function of EEG is not linear. Instead, the algorithms are based on stages. There is also the problem that the actual index ranges associated with a particular depth of anaesthesia differ in the two monitors. These aspects may, at least in part, explain the seemingly large Bland-Altman limits of agreement in the present and similar studies.^7,8^ Estimation of the hypnotic component of anaesthesia by a single numerical descriptor (index) does not include information on the temporal evolution of the index and should always be supported by inspection of the raw EEG signal. This is particularly true during the induction and emergence phases of general anaesthesia, where index values are often in a similar range, while the EEG signals are obviously different. This was demonstrated by comparing the NARCOguide and Narcotrend index during induction and emergence from anaesthesia with the relative α and δ EEG band power calculated by FFT analysis (Figure S10). Significant differences in α and δ band power are not directly reflected by a difference in either index (Tables 1 and 2).

In conclusion, the NARCOguide index can be used to monitor the hypnotic component of anaesthesia in patients undergoing general anaesthesia or sedation with propofol with a similar performance to the Narcotrend index.

## Supplementary Material

Supplemental Digital Content

## Supplementary Material

Supplemental Digital Content

## Supplementary Material

Supplemental Digital Content

## Supplementary Material

Supplemental Digital Content

## Supplementary Material

Supplemental Digital Content

## Supplementary Material

Supplemental Digital Content

## Supplementary Material

Supplemental Digital Content

## Supplementary Material

Supplemental Digital Content
