# Supplementary material for: The NARCOguide index – a novel parameter for monitoring depth of hypnosis during anaesthesia/sedation with propofol: A comparison study with the Narcotrend index
Source: Eur J Anaesthesiol Intensive Care. 2024 Jul 18;3(4):e0057. doi: 10.1097/EA9.0000000000000057 (PMC11798396; doi:10.1097/EA9.0000000000000057)
Supplement: Supplemental Digital Content [file ejaic-3-e0057-s001.pdf]

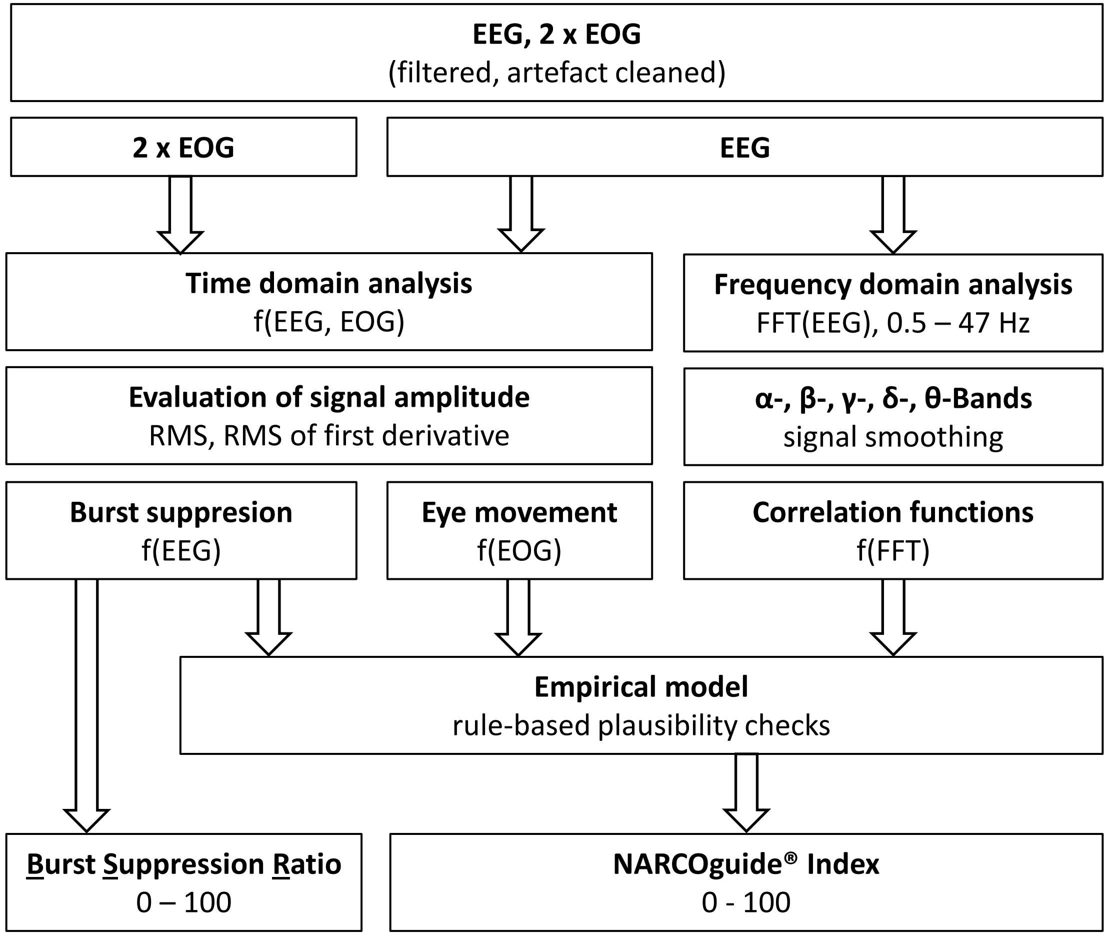

Figure S1: NARCOguide® algorithm flowchart. 2 x EOG, EOG (left channel) and EOG (right channel); RMS, root mean square.
