# Supplementary figures and images for: The NARCOguide index – a novel parameter for monitoring depth of hypnosis during anaesthesia/sedation with propofol: A comparison study with the Narcotrend index
Source: Eur J Anaesthesiol Intensive Care. 2024 Jul 18;3(4):e0057. doi: 10.1097/EA9.0000000000000057 (PMC11798396; doi:10.1097/EA9.0000000000000057)

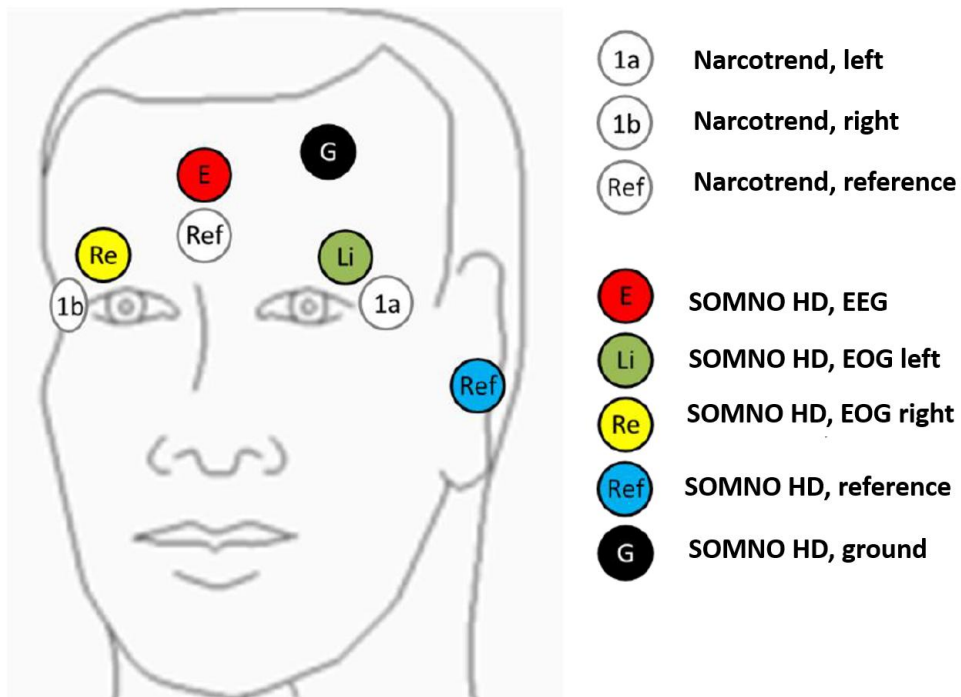

Figure S3: Application plan for head electrodes for Narcotrend® (white) and SOMNO HD (coloured).

Supplement: Supplemental Digital Content [file ejaic-3-e0057-s003.pdf]
