# Supplementary material for: The NARCOguide index – a novel parameter for monitoring depth of hypnosis during anaesthesia/sedation with propofol: A comparison study with the Narcotrend index
Source: Eur J Anaesthesiol Intensive Care. 2024 Jul 18;3(4):e0057. doi: 10.1097/EA9.0000000000000057 (PMC11798396; doi:10.1097/EA9.0000000000000057)
Supplement: Supplemental Digital Content [file ejaic-3-e0057-s004.pdf]

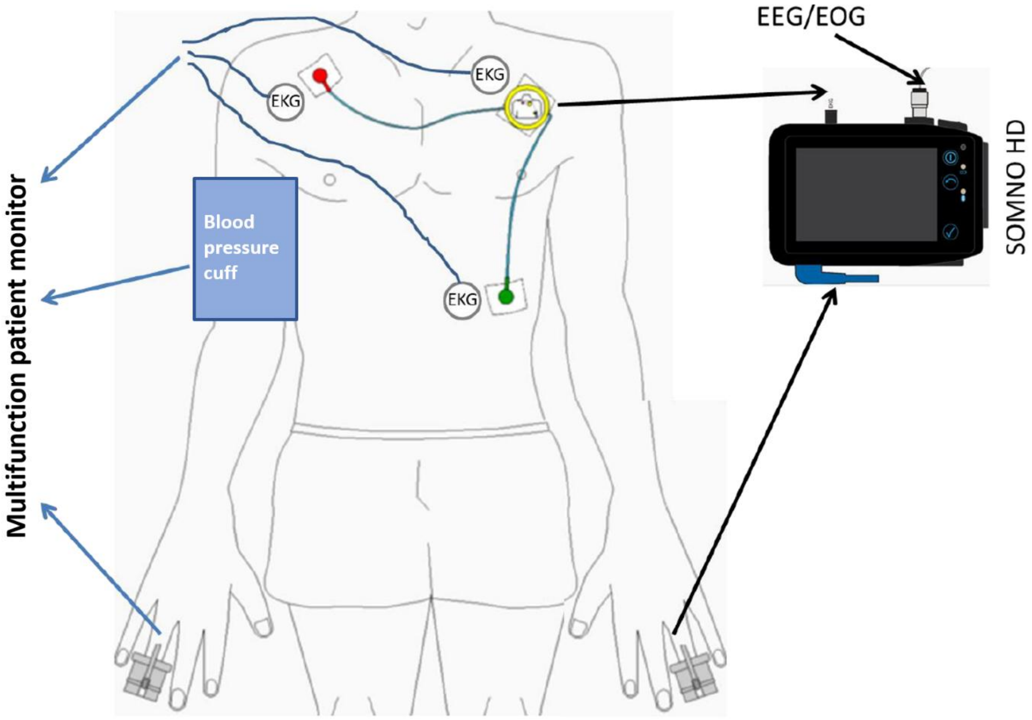

Figure S4: Application plan for ECG electrodes and finger clip sensors. ECG electrodes are applied side by side for patient monitor and SOMNO HD. Finger clip sensor and blood pressure cuff on one arm for patient monitor. Finger clip sensor on the OTHER arm for SOMNO HD. The head electrodes for the SOMNO HD are connected to the headbox and this to the headbox socket of the SOMNO HD.
