# Supplementary material for: The NARCOguide index – a novel parameter for monitoring depth of hypnosis during anaesthesia/sedation with propofol: A comparison study with the Narcotrend index
Source: Eur J Anaesthesiol Intensive Care. 2024 Jul 18;3(4):e0057. doi: 10.1097/EA9.0000000000000057 (PMC11798396; doi:10.1097/EA9.0000000000000057)
Supplement: Supplemental Digital Content [file ejaic-3-e0057-s005.pdf]

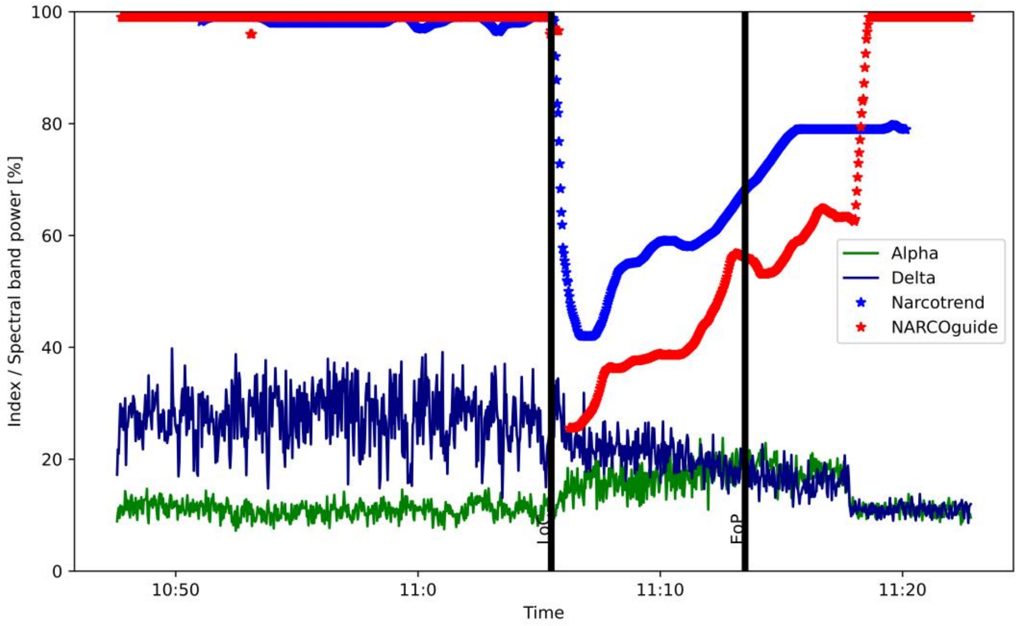

Figure S9: Example measurement (taken from OS group) illustrating the typical evolution of index values and relative band power of the α and δ spectral bands during general anaesthesia with propofol. LoC = loss of consciousness, EoP = end of propofol infusion.
