# Supplementary material for: The NARCOguide index – a novel parameter for monitoring depth of hypnosis during anaesthesia/sedation with propofol: A comparison study with the Narcotrend index
Source: Eur J Anaesthesiol Intensive Care. 2024 Jul 18;3(4):e0057. doi: 10.1097/EA9.0000000000000057 (PMC11798396; doi:10.1097/EA9.0000000000000057)
Supplement: Supplemental Digital Content [file ejaic-3-e0057-s006.pdf]

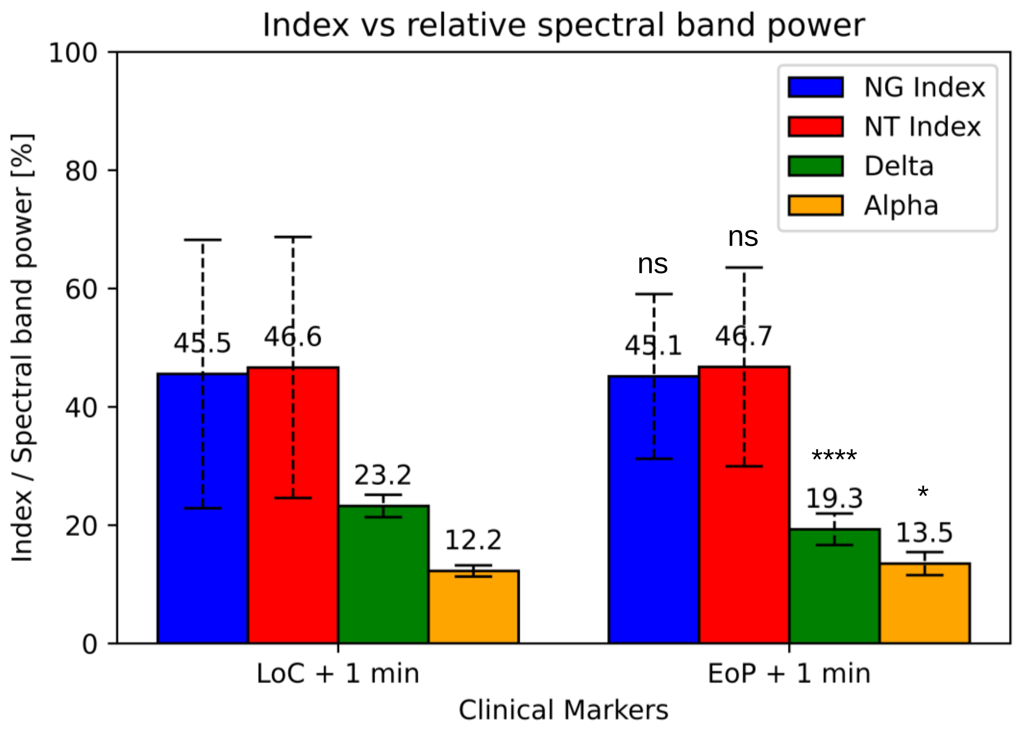

Figure S10: Comparison of the NARCOguide® (NG) index, Narcotrend® (NT) index, relative δ-band power and relative α-band power between induction (1 min after loss of consciousness (LoC + 1 min)) and emergence from anaesthesia (1 min after end of propofol infusion (EoP + 1 min)). Bars represent means ± standard deviation; P values were calculated using T test and asterisks denote statistical significance compared to (LoC + 1 min) with $P > 0.05$ ≙ not significant (ns), $P \leq 0.05$ ≙ *, $P \leq 0.01$ ≙ **, $P \leq 0.001$ ≙ ***, $P \leq 0.0001$ ≙ **.**
