# Supplementary material for: The NARCOguide index – a novel parameter for monitoring depth of hypnosis during anaesthesia/sedation with propofol: A comparison study with the Narcotrend index
Source: Eur J Anaesthesiol Intensive Care. 2024 Jul 18;3(4):e0057. doi: 10.1097/EA9.0000000000000057 (PMC11798396; doi:10.1097/EA9.0000000000000057)
Supplement: Supplemental Digital Content [file ejaic-3-e0057-s007.pdf]

Table S1: Dataset composition; entire study population.

	Total (N = 40)		Male (N = 27)		Female (N = 13)	
	**Mean**	**SD**	**Mean**	**SD**	**Mean**	**SD**
**Age** (years)	42.3	15.0	46.9	13.8	34.6	13.2
**Height** (cm)	176.2	9.7	180.1	8.8	168.0	6.0
**Weight** (kg)	83.3	19.5	86.6	13.1	78.5	27.1

SD = standard deviation

Table S2: Dataset composition; Patients undergoing oral surgery (OS).

	Total (N = 17)		Male (N = 7)		Female (N = 10)	
	**Mean**	**SD**	**Mean**	**SD**	**Mean**	**SD**
**Age** (years)	30.8	12.3	35.2	12.9	30.1	11.4
**Height** (cm)	171.6	8.5	174.5	9.2	166.2	2.9
**Weight** (kg)	72.9	14.8	78.5	17.4	68.5	10.4

SD = standard deviation

Table S3: Dataset composition; Patients undergoing procedural sedation (PS).

	Total (N = 23)		Male (N = 20)		Female (N = 3)	
	**Mean**	**SD**	**Mean**	**SD**	**Mean**	**SD**
**Age** (years)	51.9	8.7	52.2	9.1	49.8	4.5
**Height** (cm)	179.5	9.4	180.3	9.1	174.0	9.1
**Weight** (kg)	92.2	17.9	89.3	9.8	112.0	36.9

SD = standard deviation
