# Supplementary material for: The NARCOguide index – a novel parameter for monitoring depth of hypnosis during anaesthesia/sedation with propofol: A comparison study with the Narcotrend index
Source: Eur J Anaesthesiol Intensive Care. 2024 Jul 18;3(4):e0057. doi: 10.1097/EA9.0000000000000057 (PMC11798396; doi:10.1097/EA9.0000000000000057)
Supplement: Supplemental Digital Content [file ejaic-3-e0057-s008.docx]

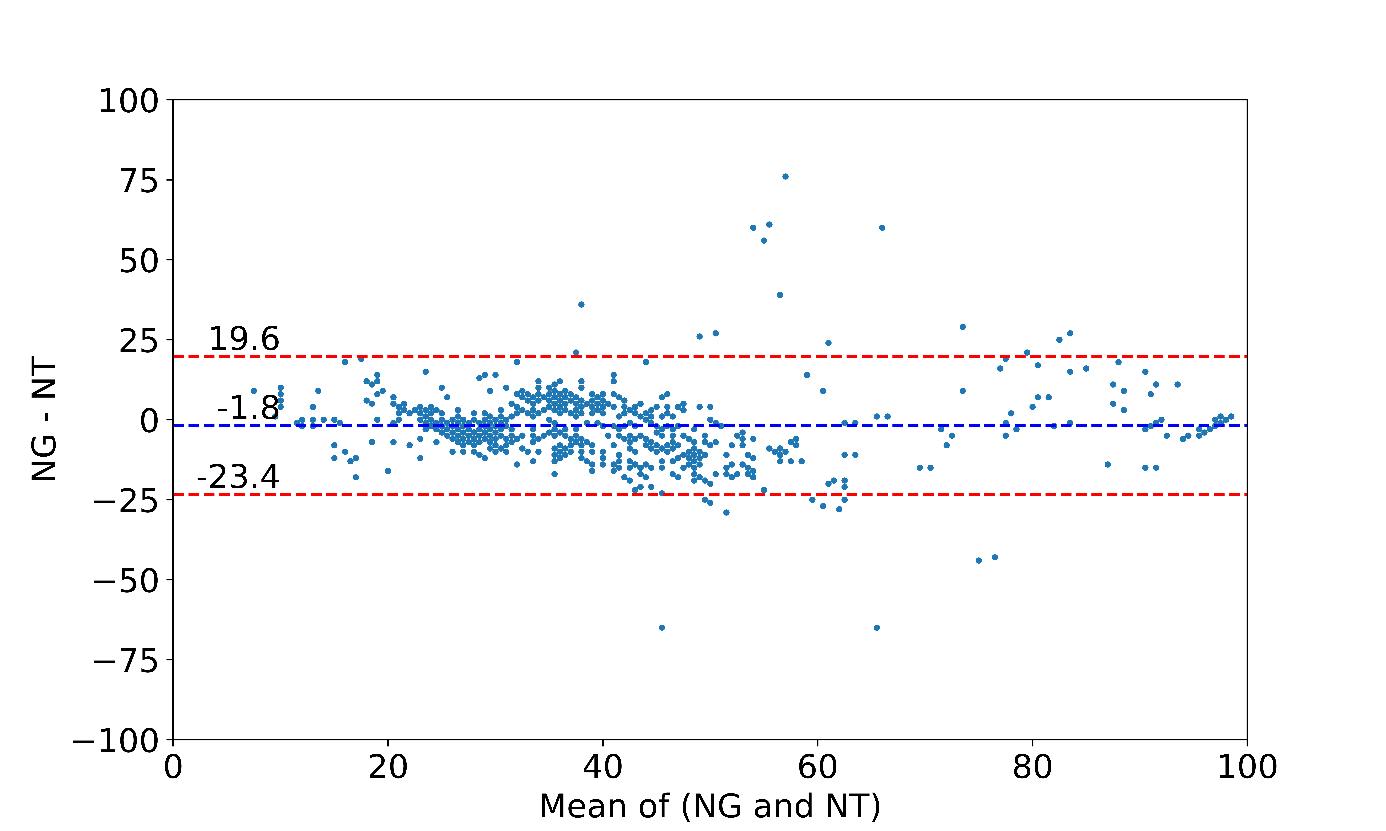

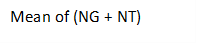


Figure S5: Bland-Altman plot for the NARCOguide® and Narcotrend® index values for patients undergoing OS. Values pooled over 1 min intervals (n = 730). Blue line represents the mean of the difference, red lines represent the mean of the difference ± 1.96 standard deviations.


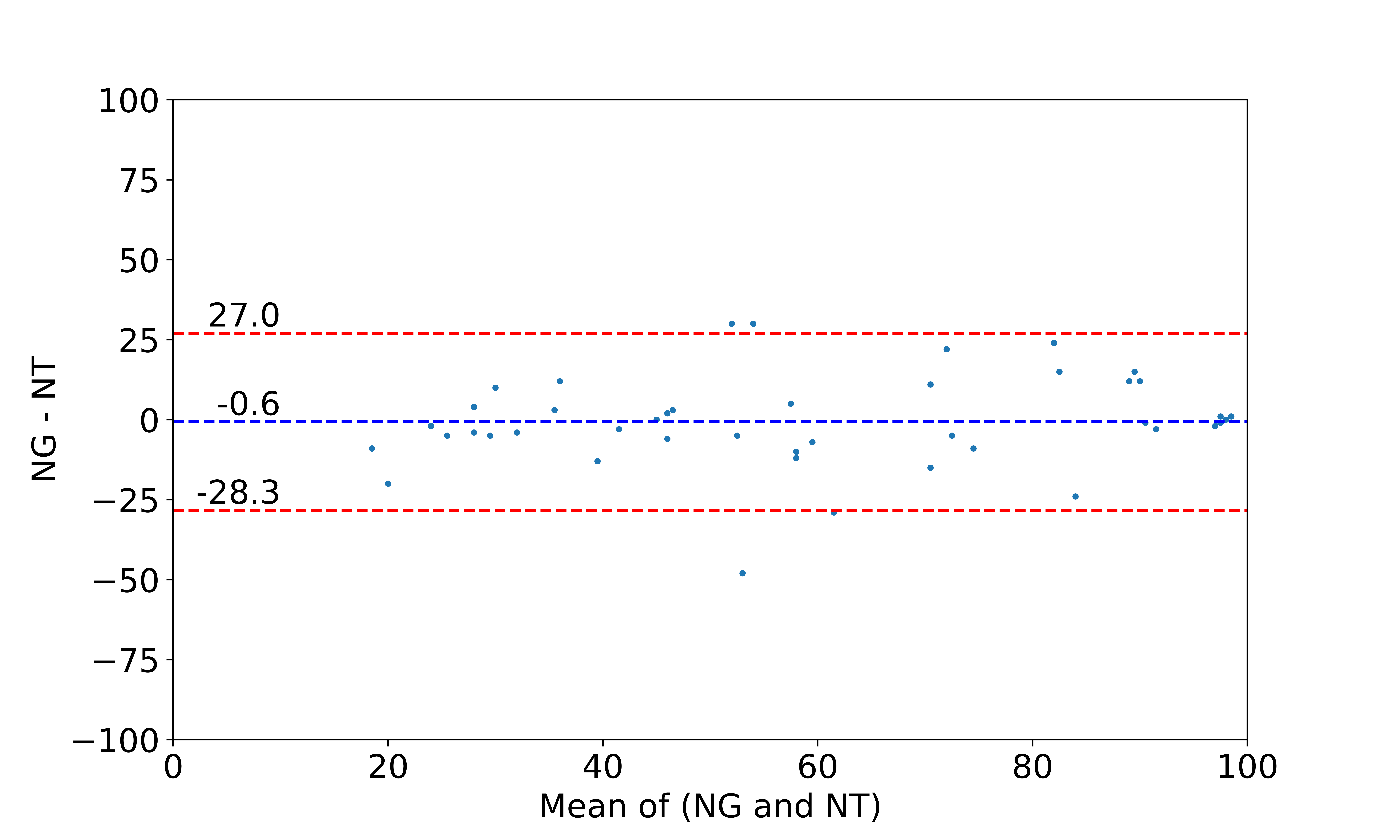

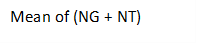


Figure S6: Bland-Altman plot for the NARCOguide® and Narcotrend® index values based on clinical markers documented by the anesthesiologist for patients undergoing OS. Values pooled over 1 min intervals (n = 46). Blue line represents the mean of the difference, red lines represent the mean of the difference ± 1.96 standard deviations.


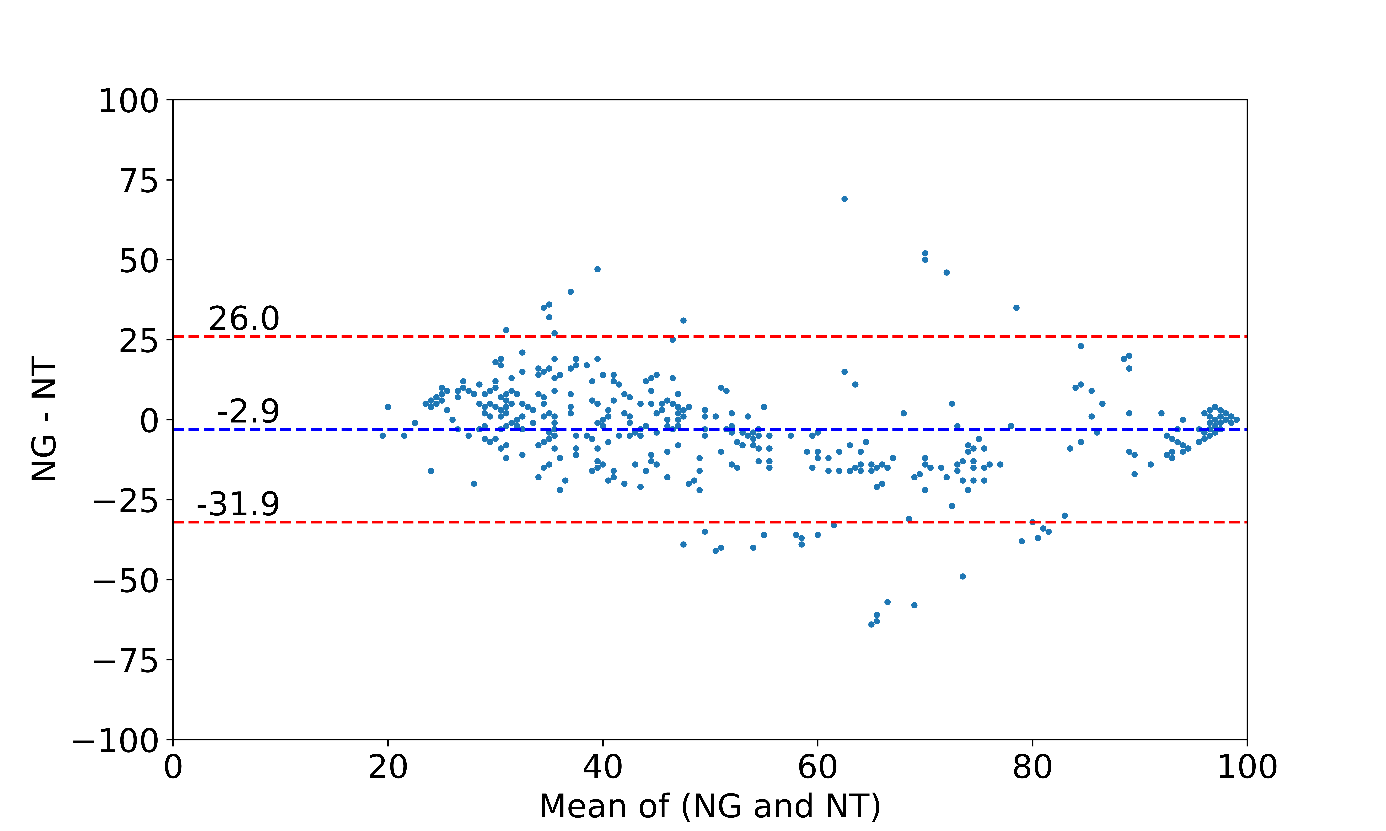

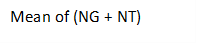


Figure S7: Bland-Altman plot for the NARCOguide® and Narcotrend® index values for patients undergoing PS. Values pooled over 1 min intervals (n = 479). Blue line represents the mean of the difference, red lines represent the mean of the difference ± 1.96 standard deviations.


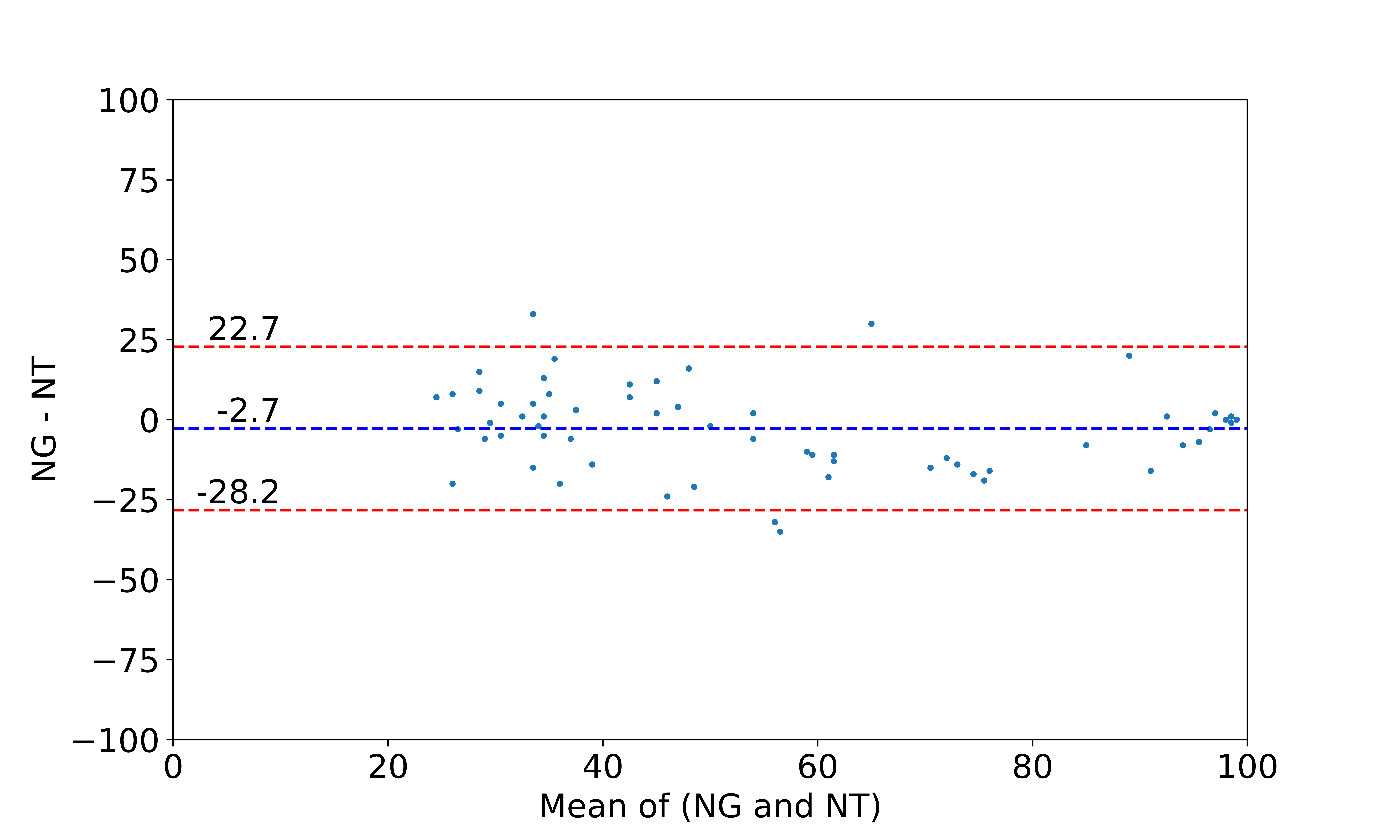

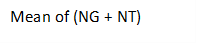


Figure S8: Bland-Altman plot for the NARCOguide® and Narcotrend® index values based on clinical markers documented by the anesthesiologist for patients undergoing PS. Values pooled over 1 min intervals (n = 65). Blue line represents the mean of the difference, red lines represent the mean of the difference ± 1.96 standard deviations.
